# Optimization of Mechanical Properties of Eco-Friendly Mortar Containing Wood Ash and Nano Silica Using Response Surface Methodology and Artificial Neural Networks

**DOI:** 10.3390/nano16120717

**Published:** 2026-06-10

**Authors:** Abiodun Akinwale, Walied A. Elsaigh, Akeem Ayinde Raheem

**Affiliations:** 1 Department of Civil Engineering & Environmental Engineering and Building Science, Florida Campus, University of South Africa, Johannesburg 1709, South Africa; hussiwam@unisa.ac.za; 2 Department of Building, Ladoke Akintola University of Technology, Ogbomosho 4000, Oyo State, Nigeria; aaraheem@lautech.edu.ng

**Keywords:** wood ash, nanosilica, compressive strength, flexural strength, artificial neural network (ANN), response surface methodology (RSM)

## Abstract

As the demand for sustainable construction materials grows, wood ash and nanosilica have emerged as promising components for eco-friendly mortars, whose optimization requires advanced analytical techniques capable of capturing their complex linear and nonlinear interactions, making frameworks such as response surface methodology and artificial neural networks essential for effective mix design. This study examines the mechanical performance of eco-friendly mortar incorporating wood ash (WA) as a partial cement replacement and nanosilica solution (NSS) as a strength-enhancing additive, with the aim of optimizing compressive and flexural behaviour. Wood ash was substituted at levels of 5–25%, while NS (0.265 moL^−1^) was substituted at levels of 0–1.7%. Twenty-one mortar samples were produced and tested at multiple curing ages. Two modelling techniques, response surface methodology (RSM) and artificial neural networks (ANNs), were employed to evaluate the individual and interactive effects of WA and NSS on strength development at curing ages of 28 and 180 days. While RSM provided insight into factor significance and linear interactions, ANN more effectively captured nonlinear behaviour, achieving superior predictive accuracy (R^2^ = 1.000 for 28-day strength). Experimental results revealed that nanosilica substantially enhanced strength up to an optimal dosage of approximately 2.5 g, beyond which performance declined due to particle agglomeration or matrix over-refinement. In contrast, higher WA contents produced strength reductions attributable to dilution effects. Optimization showed that mixtures containing low WA (≤30 g) combined with moderate NSS (2.0–2.5 g) exhibited the highest mechanical performance. Collectively, the findings confirm that ANN-based models outperform RSM and multilinear regression, underscoring their effectiveness for mix design optimization and performance forecasting in sustainable cementitious systems.

## 1. Introduction

The construction industry faces escalating sustainability pressures, with concrete second only to water in global consumption and responsible for roughly 8–10% of CO_2_ emissions, largely from cement production [1,2,3,4]. Coupled with depletion of natural resources and mounting waste streams, this has accelerated the search for eco-friendly alternatives to conventional concrete constituents [5,6]. Two promising supplementary cementitious materials (SCMs) are wood ash (WA), a biomass-combustion by-product that valorizes waste within circular economy principles, and nanosilica (NS), whose high specific surface area and reactivity can refine microstructure and boost mechanical performance [7,8,9,10,11,12]. Yet, optimizing mixes that incorporate multiple sustainable ingredients remains challenging due to nonlinear interacting effects among constituents on fresh and hardened properties; traditional trial-and-error is slow, costly, and often misses these interactions, motivating data-driven modeling via response surface methodology (RSM) and artificial neural networks (ANNs) [13,14,15,16,17].

Over the past two decades, WA has been explored as a partial cement replacement with typically weaker pozzolanic activity than fly ash but meaningful contributions through both secondary reactions and filler effects [18,19]. Several studies report viable replacement levels on the order of 10–20% without major losses in mechanical performance when WA is properly characterized and processed [12,20]. Composition varies by fuel type and combustion conditions; higher SiO_2_/Al_2_O_3_ contents generally improve pozzolanic potential [21,22]. Beyond waste diversion, life-cycle assessments indicate carbon footprint reductions of up to 15% at optimal dosages [23,24]. Practical challenges include variability, workability penalties, and unburnt carbon that may depress strength or entrain air [10,12]. Nevertheless, up to 15% of cement may be substituted with wood ash without compromising mortar efficacy [25,26,27]. In a study conducted by [28], the proposed wood ash content should not exceed 15% for optimal concrete performance.

NS improves concrete through a dual mechanism: rapid pozzolanic reaction (accelerated chemical combination of reactive amorphous silica-aluminium) with Ca(OH)_2_ and nano-scale packing that densifies the matrix and refines the pore system [29,30]. Reported compressive strength gains of up to 25% are common near 2–3% (by binder) when dispersion is well controlled [31,32]. Performance depends on particle size, surface chemistry, and dispersion method; agglomeration above optimal contents can erode benefits and reduce workability [33,34]. Reviews consistently document microstructural densification, reduced permeability, and durability gains attributable to NS-driven nucleation and pore refinement [35,36,37]. Other studies have also examined the potential use of NS in other types of concrete. Aydin et al. [38] reported that self-compacting concrete containing 2% NS, 0.08 percent nano-carbon, and 40% fly ash (FA) performed more effectively than an NS-free admixture. Naniz and Mazloom concluded that adding NS up to 3% of the cement weight improved the fresh and hardened properties of concrete with different water-to-binder ratios [39]. NS doses of up to 2–3% could enhance the material’s mechanical properties and durability, pursuant to an evaluation of the effects of nanosilica in concrete by [31]. This could be attributed to its pozzolanic action, filling effect, and refined pore structure. Compressive strength increases with NS content, and the NS functions as an activator to promote hydration. However, if the NS dosage increases by more than 3%, the strength may be reduced. Higher dosages (i.e., 3% or more) may eventually affect the engineering attributes due to the agglomeration of the NS particles, resulting in microcracking, excessive porosity, and decreased compressive strength.

Combining WA and NS can yield complementary effects: NS supplies abundant nucleation sites and early-age reaction kinetics, while WA contributes longer-term pozzolanic reactions and filler action, jointly enhancing microstructure when proportioned appropriately [40,41,42]. However, the interaction is complex—spanning chemical, physical, and rheological domains—and requires systematic optimization to balance strength, durability, and workability.

To that end, RSM offers an efficient statistical framework to map factor effects and interactions with relatively few experiments, delivering predictive polynomials and significance analyses well suited to multi-variable mix design [43,44,45,46,47]. Recent sustainable-concrete studies have used RSM to optimize strength, workability, and durability with good fidelity and actionable operating windows [48,49,50,51]. ANNs, in turn, capture strongly nonlinear relationships without imposing parametric forms, often achieving higher predictive accuracy when trained on sufficiently rich datasets with appropriate architectures and regularization [52,53,54,55]. Comparative surveys report ANNs outperforming traditional regressions for strength prediction in complex binders, provided data quality and model design are adequate [56,57,58]; R^2^ values >0.95 for compressive strength prediction in mixes with multiple SCMs are frequently reported [59,60,61,62]. Similarly, evidence from comparative studies [63] demonstrates that optimized combinations of wood ash (10–20%) cement replacement and nanosilica (1–2% by weight) can achieve comparable or superior performance to conventional supplementary cementitious materials (SCMs) such as fly ash and silica fume. Key findings include the following: (1) nanosilica additions of 1–2% enhance compressive strength by 7.8–13.5% at early ages and improve durability through pore refinement and reduced chloride penetration (RCPT), showing an up to 57.8% reduction in RCPT values [64]; (2) wood ash at optimal replacements levels (10–20%) maintains acceptable mechanical performance while offering significant carbon footprint reductions [65]; (3) synergistic combinations of wood ash with nanosilica demonstrate enhanced performance compared to either additive alone, with compressive strengths reaching 27.53–35 MPa at 90 days [66]; and (4) durability improvements include reduced water absorption, enhance sulfate resistance, and refined microstructure [67]. These findings support the viability of eco-friendly mortar formulations as sustainable alternatives in construction applications.

Despite this progress, key gaps persist: (i) limited studies that co-optimize WA and NS (and other SCMs) within an integrated design space; (ii) limited standardization of testing protocols and performance criteria for greener binders; (iii) sparse long-term durability data across environmental exposures; and (iv) few head-to-head comparisons of RSM versus ANNs for sustainable-concrete optimization [68,69,70,71,72,73,74]. Addressing these gaps, the present study investigates eco-friendly concrete with WA and NS using both RSM and ANNs to (i) quantify effects on compressive and flexural strengths at multiple curing ages, (ii) compare the predictive/optimization performance of RSM and ANNs in a shared dataset, (iii) identify optimal WA–NS combinations that maximize mechanical performance while advancing sustainability goals, and (iv) validate predictions and distill practical guidelines for implementation.

This study advances current understanding of nanosilica–wood ash cement composites by integrating experimental optimization with state-of-the-art machine learning techniques. By demonstrating the markedly superior predictive performance of Optuna-optimized ANN models compared with traditional MLR and RSM approaches, this work addresses key limitations in prior research that relied predominantly on linear empirical models. The investigation further establishes precise mixture boundaries, identifying an optimal nanosilica dosage and a critical wood ash limit, thus offering clear, experimentally validated guidelines that have not been previously reported. Through the combined use of multi-objective optimization, comparative modeling, and mechanistic interpretation, this study lays the foundation for a more data-driven and sustainable approach to designing high-performance cementitious composites.

## 2. Methodology

### 2.1. Materials and Methods

#### 2.1.1. Chemical and Physical Properties of Materials

**Figure 1:**
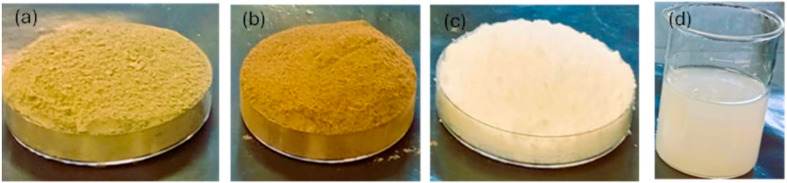
Materials used: (**a**) CEM I 52.5 N; (**b**) wood ash; (**c**) nanosilica powder, (**d**) nanosilica solution prepared in distilled water. Adapted from a prior study [40].

The chemical and physical properties of the materials used in this study are summarized in Table 1. Ordinary Portland cement conforming to SANS 50197-1 (CEM I 52.5 N) served as the primary binder. Wood ash was sourced from Blackwattle tree residues collected from commercial caterers in Johannesburg. The ash was oven-dried and sieved prior to use to ensure consistency in particle size and quality. The nanosilica employed in this study was supplied by Sigma Aldrich Boksburg, (Boksburg, South Africa). According to its Certificate of Analysis, the nanosilica contained 92.1% SiO_2_, 6.1% carbon, and 1.8% nitrogen, with an average particle diameter of <150 µm and a pore size of approximately 6 nm. The materials used in the experimental study are presented in Figure 1.

**Figure 2:**
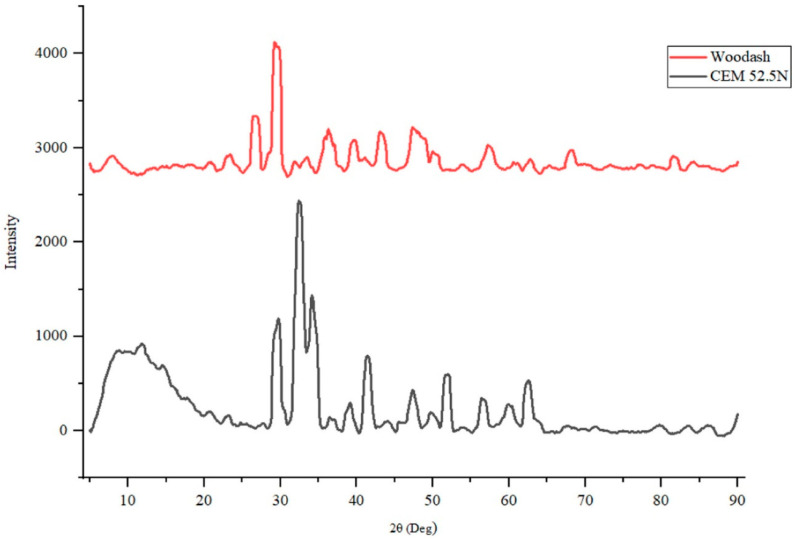
XRD patterns of wood ash and CEM I 52.5 N showing lower crystallinity in wood ash relative to cement. Adapted from a prior study [40].

The wood ash satisfied the pozzolanic oxide requirement, with the combined SiO_2_ + Al_2_O_3_ + Fe_2_O_3_ content measured at 93.67%, exceeding the minimum 70% threshold, as shown in Table 1. X-ray diffraction (XRD) analysis further confirmed the relative crystallinity of the materials. Cement exhibited sharper and more intense peaks, indicative of a higher crystalline phase. In contrast, the wood ash displayed broader diffraction humps characteristic of an amorphous-rich structure, with its most pronounced peak occurring at 2θ < 30°. This amorphous nature is consistent with the pozzolanic reactivity of wood ash and supports its potential to partially replace cement in construction applications. Cement peaks were predominantly observed between 26° and 35°, as illustrated in Figure 2.

#### 2.1.2. Mix Design and Preparation

A nanosilica solution was prepared by dissolving 3.5 g of SiO_2_ powder in 13.201 L of distilled water as reported in a prior study [40]; this yielded an approximate concentration of 0.265 mol·L^−1^ and was used as a percentage of the mixing water to ensure consistent dispersion during batching. Potable tap water was used for all mixes. Mortar samples followed EN 196-1 proportions, batching 450 ± 2 g cement, 225 ± 1 g water, and 1350 ± 5 g CEN standard sand per prism set. Wood ash replaced cement at 5, 10, 15, 20, and 25% by volume. Nanosilica was dosed at nominal 0, 0.6, 1.1, and 1.7% by binder volume and introduced as part of the mixing water to promote early dispersion. In total, twenty-one mixes were produced, with a water-to-binder ratio spanning 0.50–0.67 as determined by the fixed mass method and constant water content. Mix identities and exact batch masses are listed in Table 2.

The mixing sequence was standardized to minimize variability and ensure comparable dispersion of nanosilica. Water containing the nanosilica dose was combined with cement at time zero and mixed at low speed for 30 s. Standard sand was introduced gradually between 30 and 60 s with continuous mixing, followed by an additional 30 s at low speed to reach 90 s. A 30 s stop was used for bowl scraping, then the mix rested from 120 to 180 s; finally, it was mixed at high speed from 180 to 240 s to achieve a homogeneous mortar suitable for casting.

#### 2.1.3. Casting, Curing, and Testing

Mortar prisms measuring 40 × 40 × 160 mm were cast in steel moulds. Specimens remained in the laboratory for 24 h and were then demoulded and cured in water until the designated test ages of 3, 7, 14, 28, 90, and 180 days. Mechanical tests followed SANS 5864. Flexural strength was determined by three-point bending on the full prism; compressive strength was measured on the two halves from the flexural test. All results represent the average of three specimens per age and mix. Testing was performed on a Toni Technik machine rated to 300 kN, with the flexural and compressive failure modes documented for quality control.

### 2.2. Methods

#### 2.2.1. Response Surface Methodology (RSM)

The RSM [75] was employed to analyse the experimental results related to the flexural and compressive strength of cement mortars incorporating WA and NS. This statistical approach was used to evaluate the significance and interaction effects of the input variables—WA and NS dosage—on strength development at various curing ages. The quality of the developed model was assessed using statistical indicators such as the coefficient of determination (R^2^), adjusted R^2^, and predicted R^2^. The F-value was used to determine the relative influence of each factor, with a higher F-value indicating a more significant effect on the output response. The *p*-value was used to assess the statistical significance of the model and its terms; a *p*-value less than 0.05 was considered statistically significant, indicating that the factor or model term has a meaningful effect on strength development [76]. The RSM analysis in this study was conducted using Design Expert 12 (trial version), allowing for optimization and visualization of the influence of WA and NS contents on mortar performance.

#### 2.2.2. Artificial Neural Network (ANN)

**Figure 3:**
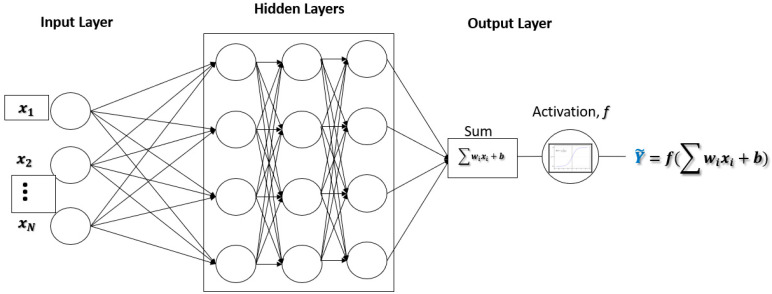
Schematic representation of an artificial neuron used in ANN modeling of strength in WA-NS blended mortars.

The Artificial Neural Network (ANN) approach was used to develop a predictive model for the strength performance of mortars containing wood ash and nanosilica. ANN is a computational model inspired by the biological neural networks of the human brain and is particularly effective for capturing nonlinear relationships among complex input variables [52,68,77]. In the context of this study, the ANN model was constructed using input features such as cement, sand, water, wood ash, nanosilica, and curing age, with the target output being either compressive or flexural strength. The ANN model was trained using 21 data points, corresponding to the 21 experimentally tested mortar mixes. Each input parameter was weighted and combined using a set of learned coefficients (weights), followed by the application of a bias term. This process is illustrated in Figure 3, which shows a schematic representation of an artificial neuron. The mathematical formulation of a single neuron is given by Equation (1) [69]:

(1) $$y=f\left(\sum_{i=1}^{n}\;\;w_{i}x_{i}+b\right)$$

where $x_{i}$ represents each input (e.g., WA, NS, water content), $w_{i}$ is the corresponding weight, b is the bias, and f is the activation function applied to produce the output.

Model training and implementation were carried out using Python 3’s scikit learn module [70], and hyperparameters were optimized [71] to improve prediction accuracy. The ANN model enabled robust prediction of mortar strength by learning from the experimental dataset and generalizing well to unseen mix compositions.

## 3. Results

### 3.1. The 28-Day Compressive Strength Analysis

**Figure 4:**
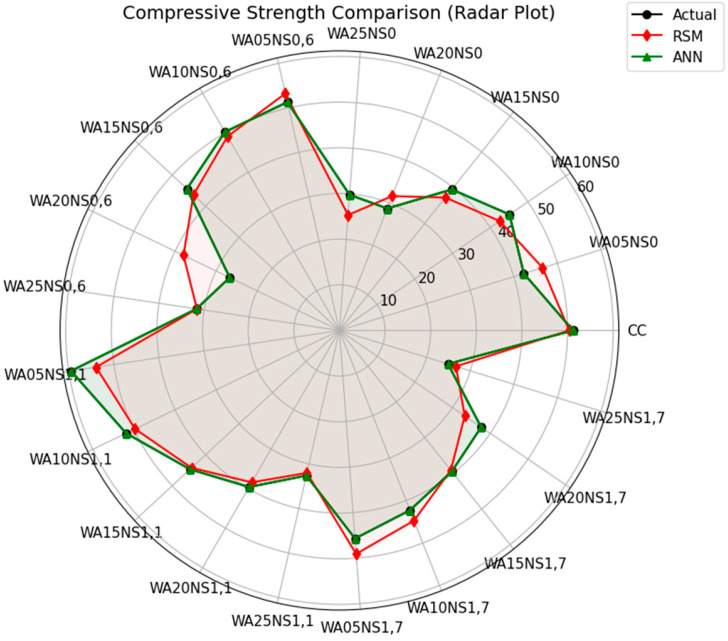
Radar plot comparing actual and predicted (RSM and ANN) compressive strength values across all mixes.

In this section, the influence of WA and NS on the 28-day compressive strength of mortar was investigated using both RSM and ANN. A summary of the design of experiments used for RSM analysis is provided in Table 3. A total of 21 mixes were tested under a user-defined quadratic model structure with randomized run order. The actual and predicted 28-day compressive strength is presented in Table 4. The radar plot comparing actual and predicted (RSM and ANN) compressive strength values across all mixes is shown in Figure 4.

The regression model for compressive strength developed using RSM is expressed in Equation (2):

(2) $$f_{cs}=-0.0012A^{2}-0.0046AB-0.2138A-1.7224B^{2}+7.3100B+50.3506$$

where A is the wood ash content (g) and B is the nanosilica content (g).

The accuracy and significance of the response surface methodology (RSM) model were evaluated using analysis of variance (ANOVA), as summarized in Table 5. The results show that nanosilica (NS) and its quadratic term exerted a statistically significant effect on compressive strength, as reflected by their low *p*-values (NS = 00.61 and NS^2^ = 0.0081). This indicates that both the linear and nonlinear contributions of NS were influential in predicting the response variable [71]. Importantly, while ANOVA provides insight into the relative statistical significance of individual predictors, the primary goal of predictive modeling methods such as RSM in optimization studies is to maximize prediction accuracy, not to establish causal inference. As emphasized by Shmueli et al. [73], *p*-values play a limited role in prediction-oriented models. Excluding variables solely because they fail to meet the conventional *p* < 0.05 threshold may actually decrease predictive performance, particularly when the model is intended to capture complex, real-world interactions.

Moreover, predictors may appear statistically non-significant due to multicollinearity, small sample sizes, or inherent variability in experimental datasets. Multicollinearity tends to inflate standard errors, resulting in weaker *p*-values even when the variables contribute meaningfully to the predictive structure of the model. Montgomery et al. [74] highlight that multicollinearity mainly affects the interpretability of individual coefficients but does not necessarily compromise the model’s ability to predict future observations. Similarly, Harrell et al. [75] argue that in small datasets, *p*-values become unstable and unreliable, and model parsimony should not come at the expense of omitting theoretically or empirically meaningful predictors. Retaining such variables enhances model stability and reduces the risk of omitted-variable bias. Consistent with this evidence, the present study retained variables that contribute to minimizing prediction error, even in cases where *p* > 0.05. This approach aligns with best practices in predictive modeling, where the emphasis is placed on overall model performance rather than isolated coefficient significance. In contrast, wood ash (WA) and the interaction term did not show statistical significance in the ANOVA results, suggesting their influence on compressive strength was comparatively weaker under the experimental conditions examined [71].

The RSM model demonstrated strong overall performance, exhibiting a high goodness of fit as indicated by the reported R^2^ and adjusted R^2^ values. These metrics confirm that the model effectively captures the variation in compressive strength attributable to the experimental factors. The close agreement between R^2^ = 0.8447, and adjusted R^2^ = 0.7929 further suggests that the model is not overfitted and maintains robust predictive capability.

**Figure 5:**
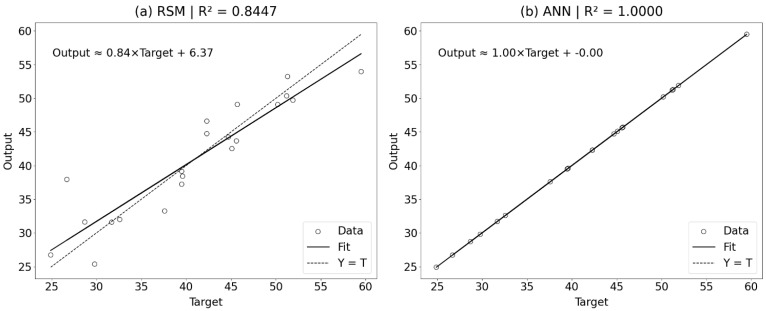
Predicted vs. actual compressive strength: (**a**) RSM model (R^2^ = 0.8447); (**b**) ANN model (R^2^ = 1.000).

Figure 5 compares the regression fits of the two models. The ANN model achieved perfect correlation with an $R^{2}=1.000$, outperforming the RSM model. This confirms that the ANN model more accurately captured the nonlinear relationships between the mix parameters and compressive strength. Ray et al. [72] likewise reported that ANN outperformed ANOVA.

**Figure 6:**
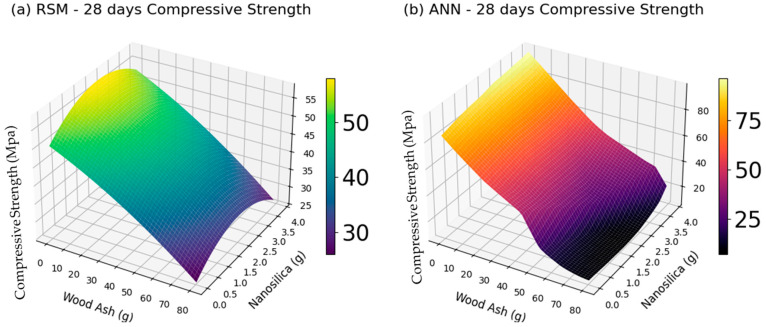
Three-dimensional surface plot of compressive strength: (**a**) RSM model; (**b**) ANN model.

The three-dimensional response surface plots shown in Figure 6 further illustrate how compressive strength varies with WA and NS. Both RSM (Figure 6a) and ANN (Figure 6b) models indicate that strength increases with NS up to a critical value (∼2 g) beyond which it begins to decline. This trend could be attributed to the beneficial pozzolanic effect of nanosilica up to an optimal dosage, beyond which particle agglomeration or excessive matrix refinement may occur [73,74]. According to İIknur et al. [78], the addition of 1 to 2% nanosilica increases compressive strength during both early and long-term strength development. The use of nanosilica at a ratio of 3% also decreases both flexural and compressive strength. It is believed that coagulation occurred with a 3% ratio, and due to the spaces, this coagulation negatively affects strength. Furthermore, a study by Abd El-Baky et al. [79] examining the effects of nanosilica on cement mortar found that nanosilica, despite being a more homogeneous binder, has fewer pores and more adhesion at the interfacial zone. In contrast, excessive dosages of NS result in more serious agglomeration of nanoparticles, resulting in a decrease in nano-effects [79,80].

Increasing WA, on the other hand, generally led to strength reduction, likely due to dilution of the cementitious matrix and pore structure disruption [81].

**Figure 7:**
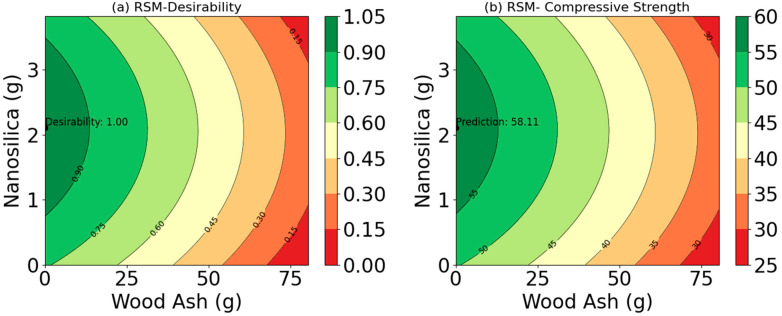
Optimization plots for compressive strength using the RSM method: (**a**) desirability, (**b**) compressive strength surface.

**Figure 8:**
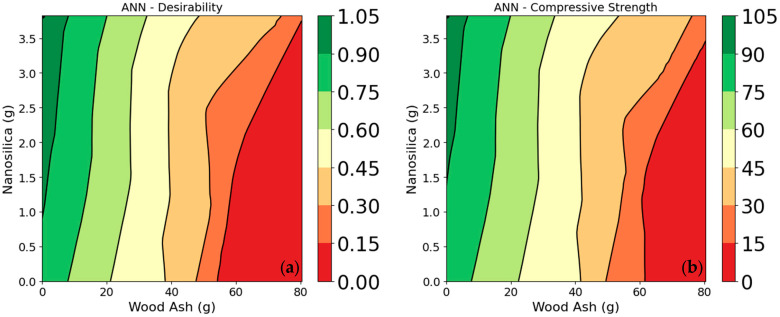
Optimization plots for compressive strength using the ANN method: (**a**) desirability, (**b**) compressive strength surface.

Figure 7 and Figure 8 present the optimization of compressive strength using RSM and ANN models, respectively. In the RSM model (Figure 7), the maximum predicted strength of 58.11 MPa was achieved at 0 g WA and 2.12 g NS, with a desirability of 1.00. This confirms that nanosilica significantly enhances strength, while excess wood ash reduces it [34]. The ANN model (Figure 8) predicted higher compressive strength (>60 MPa) under similar conditions. Its response surface shows greater nonlinearity and sharper gradients, highlighting its superior accuracy and sensitivity in capturing complex interactions [72]. Both models identify the same optimal region, but ANN demonstrates better predictive performance for mix design optimization.

### 3.2. 28-Day Flexural Strength Analysis

In this section, the effect of WA and NS on the 28-day flexural strength of concrete was investigated using both RSM and ANN. The same experimental matrix used for compressive strength (Table 3) was adopted for flexural strength analysis, under a user-defined quadratic model with randomized design.

The regression model for flexural strength using RSM is expressed in Equation (3):

(3) $$f_{fs}=-0.0004A^{2}-0.0018AB-0.0705A-0.1183B^{2}+1.0081B+6.4034$$

where *A* is the wood ash content (g) and *B* is the nanosilica content (g).

**Figure 9:**
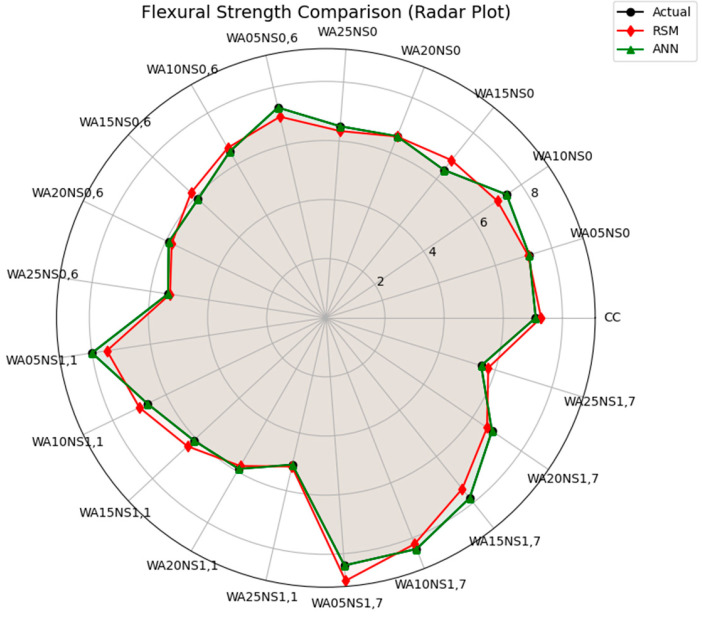
Radar plot comparing actual and predicted (RSM and ANN) flexural strength values across all mixes.

**Figure 10:**
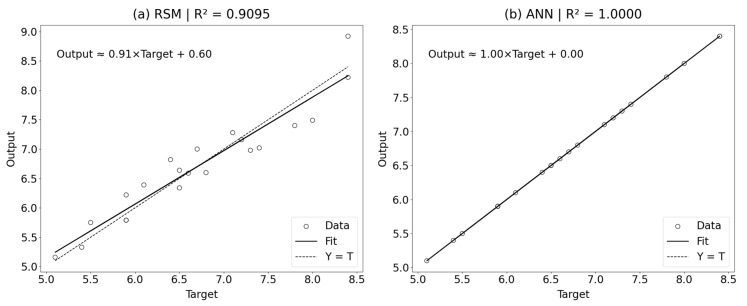
Predicted vs. actual flexural strength: (**a**) RSM model (R^2^ = 0.8621); (**b**) ANN model (R^2^ = 1.000).

The experimental values and corresponding predictions for flexural strength by both RSM and ANN are presented in Table 6. The radar plot comparing actual and predicted values is shown in Figure 9, while the regression plots are provided in Figure 10.

The ANOVA summary for the RSM model is presented in Table 7. Nanosilica and its square term were statistically significant (*p* = 0.0024 and 0.0049, respectively), while wood ash and its interaction with NS were not. The model achieved a good fit with R^2^ =0.8621 and adjusted R^2^ = 0.8163, suggesting acceptable predictive capability.

**Figure 11:**
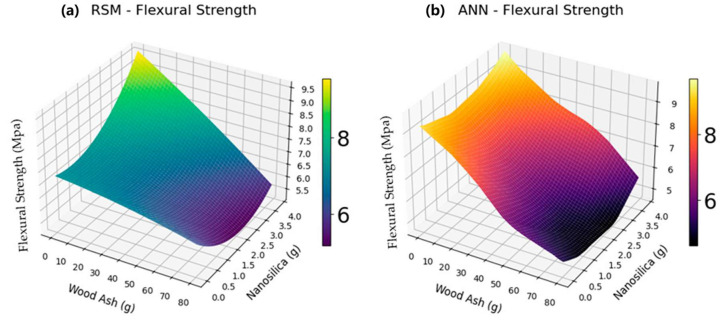
Three-dimensional surface plot of flexural strength: (**a**) RSM model; (**b**) ANN model.

The 3D response surface plots in Figure 11 illustrate the effect of WA and NS on flexural strength. Both models confirm that flexural strength increases with NS content up to about 2.3 g and then declines, indicating a saturation point. Increasing WA beyond 10 g negatively affected strength, likely due to its filler role without contributing to bonding or hydration [41].

**Figure 12:**
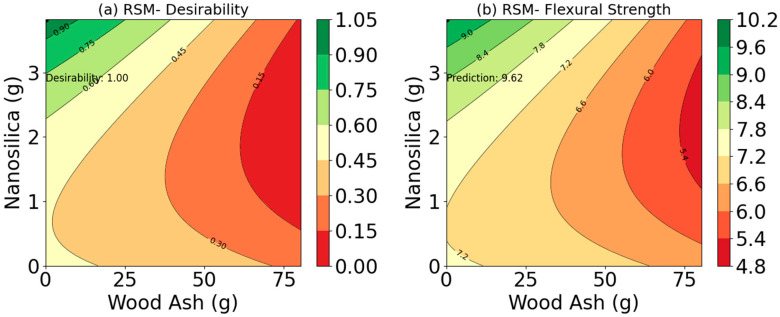
Optimization plots for flexural strength using the RSM method: (**a**) desirability and (**b**) surface flexural strength.

**Figure 13:**
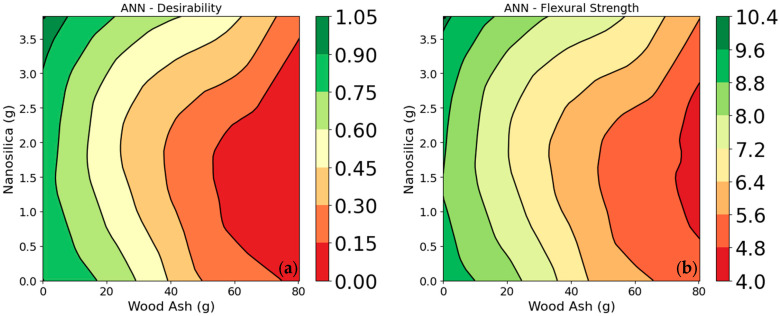
Optimization plots for flexural strength using the ANN model: (**a**) desirability; (**b**) surface flexural strength.

Figure 12 and Figure 13 show the desirability-based optimization plots. The RSM model (Figure 12) predicts a peak flexural strength of 9.18 MPa at 0 g WA and 2.3 g NS, with a desirability of 1.00. The ANN model (Figure 13) suggests slightly higher optimal strength, exceeding 10 MPa, within a similar input range, demonstrating a sharper optimal region. This supports the higher precision and robustness of the ANN model in capturing complex, nonlinear interactions in flexural strength behaviour [59].

### 3.3. 180-Day Compressive Strength Analysis

**Figure 14:**
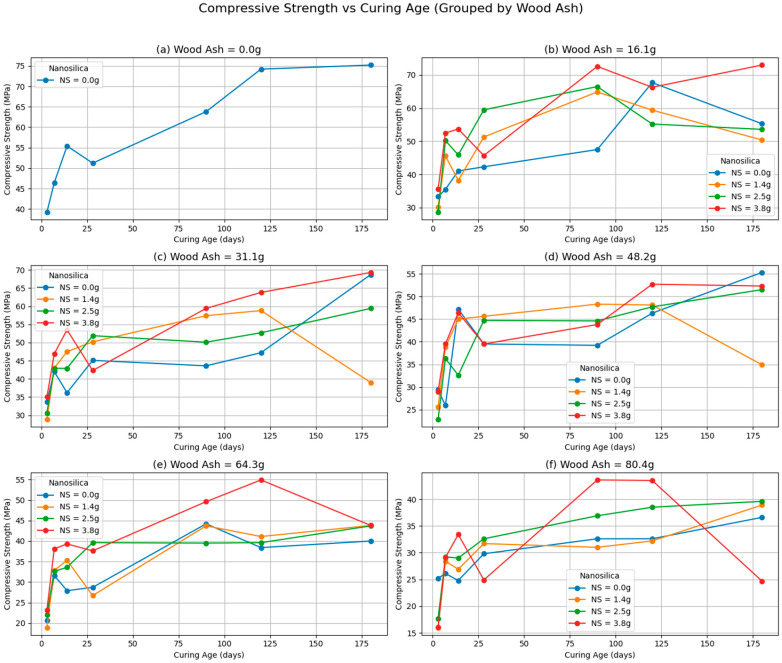
Compressive strength vs. curing age grouped by wood ash content: (**a**) WA = 0.0 g, (**b**) WA = 16.1 g, (**c**) WA = 31.1 g, (**d**) WA = 48.2 g, (**e**) WA = 64.3 g, (**f**) WA = 80.4 g. Each line represents a different nanosilica content.

**Figure 15:**
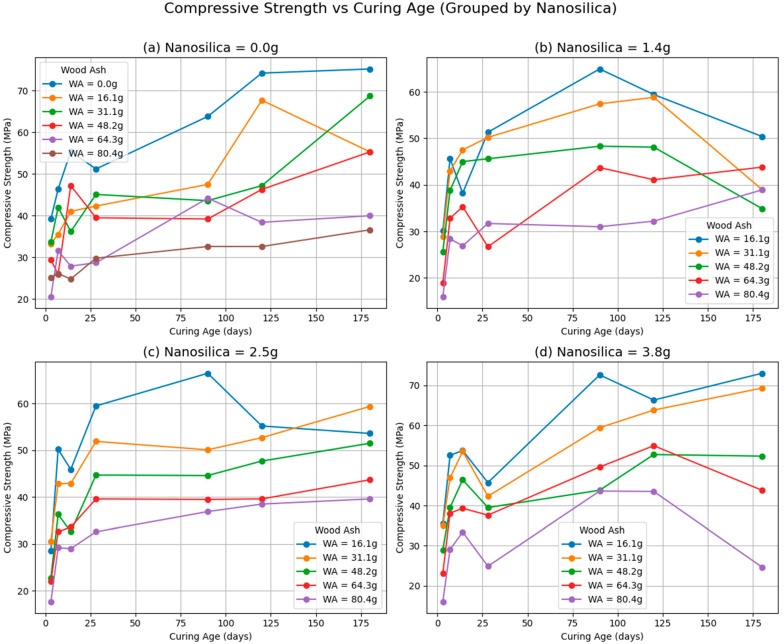
Compressive strength vs. curing age grouped by nanosilica content: (**a**) NS = 0.0 g, (**b**) NS = 1.4 g, (**c**) NS = 2.5 g, (**d**) NS = 3.8 g. Each line represents a different wood ash content.

**Figure 16:**
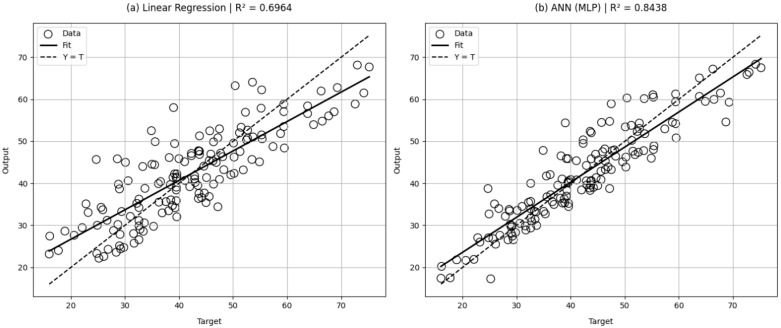
Actual vs. predicted compressive strength using: (**a**) linear regression, (**b**) artificial neural network (ANN). Solid line = model fit; dashed line = ideal fit (Y = T).

The development of compressive strength with curing age for the tested mortar mixtures is presented in Figure 14, Figure 15 and Figure 16. These results are organized in two complementary ways: grouped by wood ash content (Figure 14) and grouped by nanosilica content (Figure 15). Model predictions from multilinear regression and ANN are shown in Figure 16.

#### 3.3.1. Experimental Insights

Figure 14 illustrates the effect of curing age on compressive strength across six levels of WA content (ranging from 0.0 g to 80.4 g), with four NS contents (0.0 g, 1.4 g, 2.5 g, and 3.8 g) in each subplot. At 0.0 g WA (Figure 14a), strength development is linear and steady across curing ages, with no variation due to NS because only one NS level was tested. At 16.1 g WA (Figure 14b), the addition of NS begins to show significant early-age improvements, with NS = 2.5 g and 3.8 g resulting in notably higher compressive strength by 90 and 180 days.

For 31.1 g WA (Figure 14c), compressive strength continues to benefit from higher NS content, with NS = 3.8 g yielding the highest strength (~70 MPa) at 180 days. However, as WA increases to 48.2 g and 64.3 g (Figure 14d,e), strength development begins to stagnate or decline at lower NS dosages, emphasizing the need for sufficient NS to offset the reduced reactivity and increased porosity from high ash replacement [31,42,82,83]. The 80.4 g WA mixtures (Figure 14f) show generally reduced strength, although NS = 3.8 g still provides a clear benefit. These results highlight a synergistic relationship between WA and NS, where higher NS levels can partially counterbalance the negative impact of high ash substitution [21,36,84].

To isolate the influence of nanosilica, Figure 15 reorganizes the dataset by NS dosage, presenting four subplots corresponding to NS levels from 0.0 g to 3.8 g. Each subplot illustrates compressive strength development over time for six WA contents. At NS = 0.0 g (Figure 15a), strength gain is gradual, and mixtures with high WA contents consistently exhibit lower performance, confirming the dilutive effect of untreated ash on the cementitious matrix [22]. When NS is increased to 1.4 g (Figure 15b), a modest improvement in strength development is observed; however, performance variability persists at high WA dosages. A more pronounced enhancement emerges at NS = 2.5 g (Figure 15c), where both the magnitude and consistency of strength gain improve markedly, particularly at 90 and 180 days. The most substantial effect occurs at NS = 3.8 g (Figure 15d), where even mixtures containing high WA contents achieve appreciable long-term strength. This indicates that higher NS dosages promote pozzolanic reactions and matrix densification, counteracting the negative effects associated with elevated ash content [74].

Overall, the compressive strength results demonstrate that nanosilica is the dominant factor influencing mechanical performance. Strength increases systematically with NS content, especially at 3.8 g, due to accelerated C–S–H formation and improved microstructural packing. Wood ash exhibits a clear dosage-dependent trend: low WA levels (16–31 g) yield the highest strengths across all curing ages, whereas high WA contents (≥64 g) consistently reduce performance, likely because of clinker dilution and unreactive ash residues. Strength evolution follows typical hydration kinetics, with substantial gains between 7 and 28 days and extended long-term improvement in NS-modified mixtures. Collectively, the trends indicate that optimal compressive strength is achieved when high nanosilica dosages are combined with low wood ash contents, supporting the inclusion and relevance of these variables within the predictive RSM model.

#### 3.3.2. Predictive Modeling and Performance Comparison

The predictive accuracy of the developed models is illustrated in Figure 16. The linear regression model (Figure 16a) shows moderate agreement with experimental data (R^2^ = 0.6964), with significant scatter especially at mid-strength levels. In contrast, the ANN model (Figure 16b), tuned via Optuna, achieves stronger correlation (R^2^ = 0.8438) and better captures the nonlinear relationship between mix proportions and strength development.

The learned linear regression model is expressed as follows:

(4) $$f_{c}=19.90+0.0650\cdot \;\mathrm{Cement}\;-0.0000\cdot \;\mathrm{Sand}\;-0.0000\cdot \;\mathrm{Water}\;-0.2485\cdot \;\mathrm{WA}\;+0.7384\cdot \;\mathrm{NS}\;+0.1030\cdot \;\mathrm{Age}\;$$

where $f_{c}$ is the compressive strength (MPa), and all inputs are in grams, except curing age which is in days. The equation highlights that curing age and nanosilica content positively influence strength, while higher wood ash content has a marginally negative effect when considered independently [31,32,35]. These results emphasize the limitations of linear models in capturing interaction effects and highlight the effectiveness of machine learning techniques in modeling complex material behaviour [56]. Both models benefit from incorporating curing age as a key predictor, reinforcing the importance of maturity in strength prediction [85,86,87].

### 3.4. 180-Day Flexural Strength Analysis

**Figure 17:**
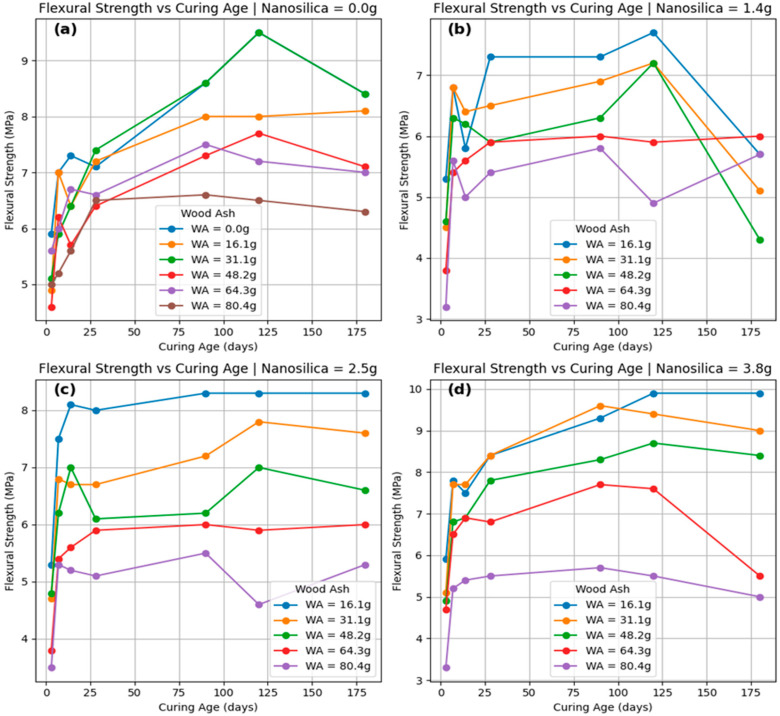
Flexural strength vs. curing age at constant nanosilica contents of (**a**) NS = 0.0 g, (**b**) NS = 1.4 g, (**c**) NS = 2.5 g, and (**d**) NS = 3.8 g for varying wood ash dosages.

**Figure 18:**
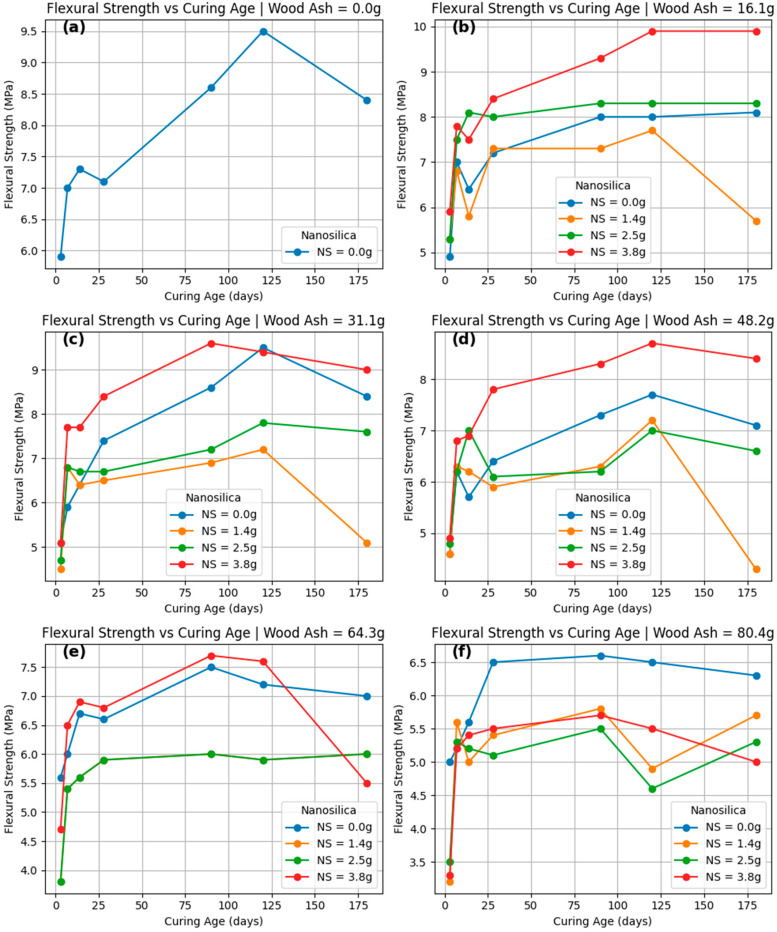
Flexural strength vs. curing age at constant wood ash contents of (**a**) WA = 0.0 g, (**b**) WA = 16.1 g, (**c**) WA = 31.1 g, (**d**) WA = 48.2 g, (**e**) WA = 64.3 g, and (**f**) WA = 80.4 g for varying nanosilica dosages.

**Figure 19:**
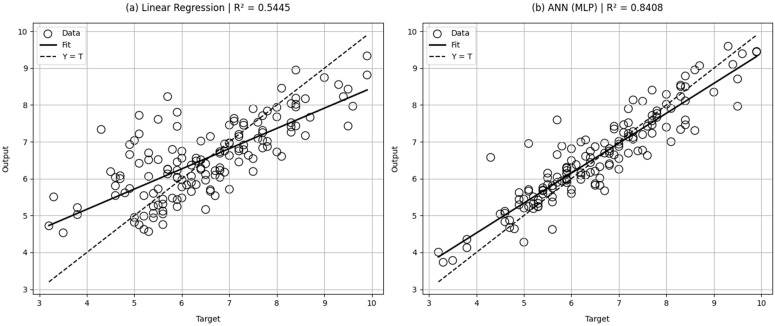
(**a**) Linear regression and (**b**) ANN (MLP) model predictions versus actual flexural strength values.

The development of flexural strength with curing age was comprehensively evaluated using both experimental analysis and predictive modeling through multilinear regression and an optimized ANN. The influence of curing age, WA, and NS on flexural performance is presented in Figure 17, Figure 18 and Figure 19.

#### 3.4.1. Experimental Insights

Figure 17 illustrates the variation in flexural strength with curing age for different WA contents at fixed NS dosages. At 0.0 g NS (Figure 17a), the flexural strength steadily increased with age, peaking around 120 days, especially for WA = 31.1 g and 48.2 g. However, strength declined at WA = 80.4 g, possibly due to dilution effects [88]. As NS was introduced at 1.4 g (Figure 17b) and 2.5 g (Figure 17c), early-age strength improved and remained relatively stable, though higher WA levels began showing diminishing returns. At 3.8 g NS (Figure 17d), strength was highest for mixes with WA between 16.1 g and 31.1 g, confirming an optimal synergy at moderate replacement levels.

Figure 18 complements this by fixing WA levels and varying NS. At 0.0 g WA (Figure 18a), flexural strength peaked at 120 days, with no benefit from NS. However, at 16.1 g WA (Figure 18b) and 31.1 g WA (Figure 18c), significant improvement occurred with 2.5 g–3.8 g NS. At 48.2 g WA (Figure 18d), this benefit persisted up to 120 days, while at higher WA levels (Figure 18e,f), flexural strength plateaued and eventually declined, indicating a threshold beyond which further WA replacement impairs mechanical gain.

#### 3.4.2. Predictive Modeling and Performance Comparison

Predictive models were trained to estimate flexural strength based on mix composition and curing age. The multilinear regression model produced the following equation:

Flexural Strength (MPa) = −7.0287 + 0.0321·Cement + 0.0000·Sand + 0.0000·Water + 0.0141·Wood Ash − 0.1684·Nanosilica + 0.0086·Curing Age(5)

This model yielded an R^2^ of 0.5445, suggesting modest predictive accuracy. In contrast, the optimized ANN model (tuned via Optuna [81]) significantly outperformed the MLR, achieving an R^2^ of 0.8408. This suggests that the nonlinear interactions among mix parameters were better captured by the ANN architecture [59,72]. Figure 19 presents parity plots comparing predicted and actual values. The linear regression model (Figure 19a) shows larger scatter, especially at higher strengths, while the ANN model (Figure 19b) follows the ideal 1:1 line more closely.

Table 8 and Table 9 show the input and response parameter levels of the factors; it can be deduced that there is a strong correlation between the input variables and the target outputs, showing a relatively low variability in the dataset. To meet the service requirement of the final product, the optimization was maximized for the compressive strength and flexural strength. For the compressive strength and flexural strength, the optimization models predict the optimal value of responses whose desirability is approximately 1.00 based on the optimization target.

## 4. Conclusions

This study demonstrated the potential of combining RSM and ANN to model and optimize the mechanical properties of mortar containing wood ash and nanosilica as an eco-friendly construction composite material. The following key conclusions were drawn.

- For 28-day compressive strength, the RSM model predicted a maximum value of 58.11 MPa at 0 g wood ash and 2.12 g nanosilica, while the ANN model estimated values exceeding 60 MPa under comparable conditions. Overall, the findings demonstrate that nanosilica substantially improves strength development, whereas higher wood ash contents reduce strength due to dilution effects and disruption of the cementitious matrix.
- For 28-day flexural strength, the RSM model predicted a maximum of 9.18 MPa at 0 g wood ash and 2.3 g nanosilica, whereas the ANN model projected values exceeding 10 MPa, thereby reinforcing the superior ability of the ANN approach to capture nonlinear patterns within the dataset.
- MLR models showed only moderate predictive accuracy, whereas Optuna-optimized ANN models achieved substantially higher R^2^ values, confirming ANN as the more reliable approach for forecasting mechanical performance.
- Optimization results indicated that a nanosilica dosage of 2.0–2.5 g consistently produced the highest mechanical performance.

This study uniquely integrates experimental optimization with Optuna-enhanced ANN modeling, demonstrating significantly higher predictive accuracy than conventional MLR and RSM methods. It further establishes precise performance thresholds identifying 2.0–2.5 g nanosilica as optimal and limiting wood ash to below 30 g, providing clear, mechanistically supported guidelines for sustainable mix design.

## 5. Suggested Future Research Studies

1. **Microstructural and Chemical Mechanisms**

Further studies should investigate the microstructural evolution and chemical interactions associated with varying nanosilica and wood ash dosages using advanced characterization techniques (e.g., XRD, SEM–EDS, FTIR). This would help clarify the mechanisms behind the observed strength trends.

2. **Durability and Long-Term Performance**

Long-term durability assessments including chloride penetration, carbonation resistance, sulfate attack, freeze–thaw cycles, and drying shrinkage are needed to determine how optimal NS and WA dosages influence performance beyond early-age strength.

3. **Broader Optimization Frameworks**

Future work could explore more advanced or hybrid optimization techniques (e.g., genetic algorithms, Bayesian optimization, particle swarm optimization) to further refine and automate mixture design targeting multiple performance objectives.

4. **Model Generalization and Transferability**

Additional datasets from different binders, aggregates, and curing regimes should be used to evaluate the generalizability of ANN models and assess their robustness across diverse mix designs.

5. **Hybrid or Physics-Informed Machine Learning Models**

Combining machine learning with mechanistic or physics-based models may enhance model interpretability and allow more accurate predictions under extrapolated conditions.

6. **Life-Cycle and Sustainability Assessments**

Given the use of wood ash as a supplementary material, future research should incorporate life-cycle assessment (LCA) and cost–benefit analysis to quantify environmental and economic impacts alongside mechanical performance.

7. **Field-Scale Validation**

Pilot-scale or field-based trials are recommended to validate laboratory-based predictions and confirm the practical feasibility of mixtures optimized using ANN models.

## Figures and Tables

**Table 1:** Chemical and physical properties of wood ash (ZAWA).

Properties		Value	Chemical Composition (%)
		SiO_2_	53.57
		Al_2_O_3_	33.98
		Fe_2_O_3_	6.12
Conventional parameters		MgO	6.23
Organic material (mg/kg)	<10	CaO	3.24
pH	12.1	MnO	1.66
		SO_3_	1.58
Physical properties		Cl	0.07
Density (kg/m^3^)	834	Na_2_O	0.00
Specific gravity	1.92	K_2_O	0.20
Mean size (µm)	0.4	TiO_2_	0.20
		P_2_O_5_	0.30
		SrO_2_	0.08
		ZrO_2_	0.96
		ZnO	2.66
		**(LOI)**	**14.2**

**Table 2:** Mix proportions for wood ash/nanosilica mortars.

Mix ID	PC (g)	Sand (g)	Water (g)	Wood Ash (g)	NS (g)
CC	450.0	1350	225	0.000	0.000
WA05NS0	427.5	1350	225	16.071	0.000
WA10NS0	405.0	1350	225	31.143	0.000
WA15NS0	385.5	1350	225	48.214	0.000
WA20NS0	360.0	1350	225	64.286	0.000
WA25NS0	337.5	1350	225	80.357	0.000
WA05NS0.6	427.5	1350	225	16.071	1.350
WA10NS0.6	405.0	1350	225	31.143	1.350
WA15NS0.6	385.5	1350	225	48.214	1.350
WA20NS0.6	360.0	1350	225	64.286	1.350
WA25NS0.6	337.5	1350	225	80.357	1.350
WA05NS1.1	427.5	1350	225	16.071	2.475
WA10NS1.1	405.0	1350	225	31.143	2.475
WA15NS1.1	385.5	1350	225	48.214	2.475
WA20NS1.1	360.0	1350	225	64.286	2.475
WA25NS1.1	337.5	1350	225	80.357	2.475
WA05NS1.7	427.5	1350	225	16.071	3.825
WA10NS1.7	405.0	1350	225	31.143	3.825
WA15NS1.7	385.5	1350	225	48.214	3.825
WA20NS1.7	360.0	1350	225	64.286	3.825
WA25NS1.7	337.5	1350	225	80.357	3.825

**Table 3:** Design summary of the RSM method.

Study Type	Design Type	Design Model	Subtype	Runs	Blocks
Response Surface	User-Defined	Quadratic	Randomized	21	No Blocks

**Table 4:** Actual and predicted 28-day compressive strength (MPa).

Test Specimen	Actual CS (MPa)	Predicted CS (RSM)	ANN Predicted (MPa)
CC	51.2	50.35	51.2
WA05NS0	42.3	46.60	42.3
WA10NS0	45.1	42.52	45.1
WA15NS0	39.5	37.23	39.5
WA20NS0	28.7	31.61	28.7
WA25NS0	29.8	25.36	29.8
WA05NS0.6	51.3	53.23	51.3
WA10NS0.6	50.2	49.06	50.2
WA15NS0.6	45.6	43.66	45.6
WA20NS0.6	26.7	37.94	26.7
WA25NS0.6	31.7	31.59	31.7
WA05NS1.1	59.5	53.96	59.5
WA10NS1.1	51.9	49.71	51.9
WA15NS1.1	44.7	44.23	44.7
WA20NS1.1	39.6	38.42	39.6
WA25NS1.1	32.6	31.99	32.6
WA05NS1.7	45.7	49.08	45.7
WA10NS1.7	42.3	44.74	42.3
WA15NS1.7	39.5	39.15	39.5
WA20NS1.7	37.6	33.25	37.6
WA25NS1.7	24.9	26.72	24.9

**Table 5:** ANOVA table for compressive strength (RSM model).

Source	Sum of Squares (SS)	df	F-Value	*p*-Value
WA	31.1778	1.0	1.7220	0.2092
NS	183.9173	1.0	10.1582	0.0061
WA^2^	7.9832	1.0	0.4409	0.5167
NS^2^	168.7324	1.0	9.3195	0.0081
WA × NS	0.5068	1.0	0.0280	0.8694
Residual	271.5787	15.0	-	-
Total $R^{2}$	0.8447	-	-	-
Adj. $R^{2}$	0.7929	-	-	-
Std. Dev.	3.5962	-	-	-
Mean	40.9714	-	-	-
C.V. (%)	8.7772	-	-	-

**Table 6:** Actual and predicted 28-day flexural strength (MPa).

Test Specimen	Actual FS (MPa)	Predicted FS (RSM)	ANN Predicted (MPa)
CC	7.10	6.40	7.10
WA05NS0	6.20	6.31	6.20
WA10NS0	5.60	5.76	5.60
WA15NS0	4.70	5.21	4.70
WA20NS0	3.20	4.30	3.20
WA25NS0	3.00	3.45	3.00
WA05NS0.6	7.40	7.14	7.40
WA10NS0.6	6.40	6.58	6.40
WA15NS0.6	5.70	6.01	5.70
WA20NS0.6	3.60	5.01	3.60
WA25NS0.6	4.00	4.11	4.00
WA05NS1.1	8.60	7.91	8.60
WA10NS1.1	7.40	7.34	7.40
WA15NS1.1	6.50	6.77	6.50
WA20NS1.1	5.10	5.76	5.10
WA25NS1.1	4.30	4.83	4.30
WA05NS1.7	6.90	7.34	6.90
WA10NS1.7	6.10	6.77	6.10
WA15NS1.7	5.10	6.20	5.10
WA20NS1.7	4.50	5.19	4.50
WA25NS1.7	3.10	4.27	3.10

**Table 7:** ANOVA table for flexural strength (RSM model).

Source	Sum of Squares (SS)	df	F-Value	*p*-Value
WA	6.2184	1	2.4146	0.1405
NS	27.4172	1	10.6411	0.0024
WA^2^	0.5801	1	0.2252	0.6410
NS^2^	23.8191	1	9.2410	0.0049
WA × NS	0.2186	1	0.0847	0.7743
Residual	38.6374	15	—	—
Total R^2^	0.8621	—	—	—
Adj. R^2^	0.8163	—	—	—
Std. Dev.	0.7586	—	—	—
Mean	5.6857	—	—	—
C.V. (%)	13.3432	—	—	—

**Table 8:** Predictive accuracy of RSM and ANN (Input).

Factor	Name	Units	Minimum	Maximum	Coded Low	Coded High	Mean	Std. Dev.
A	Wood ash	g	16,071	80,357	−1 ↔ 16	+1↔ 80,357	48,214	5.45
B	Water	g	225	225	−1↔ 225.00	+1 ↔ 225	225	0
C	Cement	g	338	427.5	−1 ↔ 338.00	+1 ↔ 428	383	63.64
D	Nanosilica	g	1.35	3825	−1 ↔ 1.35	+1 ↔ 4	2603	1.73

**Table 9:** Response.

Response	Name	Units	Maximum	Mean	Std. Dev.
R2	Compressive strength	MPa	60	59	0.71
R3	Flexural strength	MPa	10	9.59	0.29

## Data Availability

Data used in this study is part of the author’s postdoctoral research programme, which can be made available upon request.

## Abbreviations

The following abbreviations are used in this manuscript:

WA	Wood ash
NS	Nanosilica
RSM	Response surface methodology
ANN	Artificial neural networks
CS	Compressive strength
CC	Control concrete without WA and NS
